# The importance of co-morbidity and environmental risk factors for the development of migraine

**DOI:** 10.1186/1129-2377-14-S1-P28

**Published:** 2013-02-21

**Authors:** H Le, P Tfelt-Hansen, A Skytthe, KO Kyvik, J Olesen

**Affiliations:** 1 Glostrup Hospital/Danish Headache Center, Denmark; 2 University of Southern Denmark/The Danish Twin Registry, Denmark; 3 University of Southern Denmark/Institute of Regional Health Services Research, Denmark

## Introduction

Several twin studies have shown that the heritability of the migraine phenotype is approximately 40-60% [1]. The rest should, therefore, be accounted for by environmental factors. Our understanding of the risk factors of migraine remains limited despite many years of research.

## Objectives

To identify possible risk factors for the development of migraine in an adult population.

## Methods

The Danish Twin Omnibus 1994 and 2002 were questionnaire studies among almost 30,000 respectively 35,000 twin individuals [2,3]. They included questions regarding somatic disorders, lifestyle factors and socioeconomic factors. Both studies used the same validated questions to diagnose migraine. Our study population included twin individuals born between 1953 and 1976 (both inclusive) who had answered both migraine questions in 1994 and 2002. Subjects who reported having or having had migraine in 1994 were excluded.

## Results

This study comprised 13,498 subjects aged 18 to 41 years (6,513 men and 6,985 women). The 8 year risk of developing migraine was significantly increased in subjects who already had low back pain (OR: 1.3; 95%CI: 1.2-1.4). Environmental factors associated with an increased risk of development of migraine were low education (OR: 1.3; 95%CI: 1.1-1.5), hard physical workload (OR: 1.1; 95%CI: 1.0-1.2), hard physical activity (OR: 1.2; 95%CI: 1.0-1.3) and BMI < 18.5 (OR: 1.3; 95%CI: 1.1-1.6). Weekly or more often alcohol consumption had a lower risk of developing migraine compared to subjects with less frequent or no consumption of alcohol (OR: 0.6; 95%CI: 0.6-0.7).

## Conclusions

This longitudinal study comprised a large representative, non-selective sample of the Danish adult population. The main findings indicated that the development of self-reported migraine was associated with low back pain and several environmental factors. More longitudinal studies of the influence of risk factors for the development of migraine are needed.

## Competing interests

All authors declare that we have no significant competing financial, professional or personal interests.
